# Interview with Drew Weissman, 2023 Nobel Laureate in Physiology or Medicine

**DOI:** 10.20411/pai.v9i1.698

**Published:** 2024-04-16

**Authors:** Michael M. Lederman, Neil S. Greenspan

**Affiliations:** 1 Case Western Reserve University, Cleveland, Ohio

*This interview has been lightly edited for clarity

## INTRODUCTION

Drew Weissman, MD, PhD, received the 2023 Nobel Prize in Physiology or Medicine together with Katalin Karikó, PhD. Dr. Weissman received his bachelor's and master's degrees from Brandeis University, Waltham, MA, in 1981. He received his MD and PhD in 1987 from Boston University, Boston, MA, and this was followed by a residency at Beth Israel Deaconess Medical Center, Boston, MA. He then completed a fellowship at the National Institute of Allergy and Infectious Diseases under the supervision of Anthony Fauci, MD. He joined the Faculty at the University of Pennsylvania, Philadelphia, in 1997, where, in collaboration with Dr. Katalin Karikó, he explored the use of messenger RNA (mRNA) to drive heterologous gene expression in human cells. They overcame the notorious susceptibility of RNAs to degradation by packaging the mRNA in lipid nanoparticles and learned to both optimize protein expression and attenuate the inflammatory response to the exogenous RNAs by [covalently] modifying bases in the RNA sequence. This work has revolutionized immunization technology and allowed for the production of the most effective vaccines to prevent COVID-19.

## MICHAEL M. LEDERMAN, MD

Welcome to *Pathogens and Immunity*, Dr. Weismann.

## DREW WEISSMAN, MD, PHD

Thank you very much.

## NEIL S. GREENSPAN, MD, PHD

What early influences steered you to science in general and immunology in particular?

## DW

Yeah, you know, it's been so long, I don't remember the specifics. I remember, as a kid, I was always interested in math and science and engineering. And I excelled at that through elementary, middle, and high school. When I went to college, I spent summers doing basic science research in the Harvard School of Public Health and other local universities. And that really exposed me to basic science, and I fell in love with it. When I went to med school, I did an MD/PhD and primarily was interested in research.

## MML

Were there any particular books that you read or articles that you read that really got you excited?

## DW

So, there were lots of books. What really excited me the most were journal articles from the early 2000s and the early 1900s—going back to the beginning of journals. The original Watson and Crick DNA description articles, Susumu Tonegawa's description of a B cell rearrangement. There were just so many. And I was fascinated because, to me, their technology was so limited. They didn't have PCR, they didn't have all the modern things we've got, but they made such incredible breakthroughs.

## MML

So, why messenger RNA? What made you think of such a thing? Why would you do that?

## DW

My guess is it's an underdog thing. So, when I joined Kati at Penn, she had been working on RNA for 8 or 9 years and wasn't getting very far. And during her time working on RNA, there had been clinical trials using mRNA for cancer vaccines. They failed. There was no efficacy. And I think at that point, biotech and pharmaceutical companies said, “This isn't going to work; it isn't worthwhile; you don't make enough protein.” And they gave up on it. And the world pretty much lost interest in RNA. When I met Kati, I was working on vaccines. And I was working on loading dendritic cells with antigen. And wanted to try every possible way. I had DNA and peptides and viruses and proteins. I didn't have RNA. And when I met Kati at the photocopy machine, she told me she works in RNA. So, we started to work together and design immunogens to load dendritic cells as a vaccine.

## NG

Has anyone put a plaque on that photocopy machine?

## DW

The photocopy machine was, of course, replaced—that was 26 years ago. Journalists do come here and say, “We have to photograph you around a photocopy machine.” And it's really hard to find photocopy machines anymore.

## NG

Do you think the Nobel committee should have recognized, along with Katalin Karikó and yourself, the individual who first translated mRNA *in vitro*?

## DW

So, we talked with the person who ran the Nobel Prizes, and one of the things that we brought up—I don't think he was happy—was other people that we thought deserved the award. And that was one. And what he said was that there were 2 papers that came out at the same time, in 1961, describing mRNA. And then there was a series of papers, isolating hemoglobin RNA from red cells and translating it by doing *in vitro* transcription. So, there were huge numbers of people. The person that I thought should have been recognized along with us was Pieter Cullis, who invented the lipid nanoparticle.

## MML

Was there a key observation that you made early on that made you think this would actually work?

## DW

There were lots of interesting data points. As you know, in research, it's 4 steps back, and maybe 1 step forward. So, we were constantly doing experiments that didn't work. I think what helped us the most is that we tried to figure out why they didn't work. So, we understood what the mechanism wasn't. And that started to point us in the direction of what it was. There were lots of interesting findings when we could easily translate RNA in cells. We could translate RNA in mice, except the mice got sick. And all of that led us to understand what the problem was and figure out the solutions.

## MML

Every time I looked at a tube containing RNA it degraded. And so, were your colleagues and friends enthusiastic about your work? Or did they try to dissuade you from continuing?

## DW

I would go to HIV meetings in the early 2000s. And I would talk to people I knew, and some are very famous people. And I would tell them about the RNA work. And they would sit, and they would nod, and they would smile, and at the end, they would say, “You know, you really need to switch to something interesting. You're wasting your career on RNA. It's never going to go anywhere.” So, everybody tried to convince us that RNA was a fool's errand and we needed to do something else.

## MML

I think that's just wonderful. That's fantastic.

## NG

So, what do you think are the most important elements in manufacturing and delivery of the mRNA that have contributed to its success?

## DW

We're using the same synthesis procedure that was the first one invented: *in vitro* transcription using phage RNA polymerases. The procedure is better now, but it's the same enzyme system.

## NG

You're still using the phage polymerases?

## DW

Yes, and that's how Moderna and Pfizer and BioNTech and everybody else does it. What makes it expandable and inexpensive and easy, is it's a simple enzymatic reaction. There are no mammalian cells, there's no cell culture, there's no need for unknown proteins, media, serum, anything. It's a very straightforward reaction. And you can expand that reaction up easily. When J&J makes their adenovirus vaccine, they use 50,000-liter drums of CHO (Chinese hamster ovary) cells or other cells, and then they have to purify it. Moderna and Pfizer use 100-liter bioreactors and make the same number of doses or more of RNA vaccine. It's very easy to expand and produce.

## NG

That's extraordinarily interesting because it's so much more efficient as a biochemical reaction versus a biological reaction.

## MML

One hundred liters, I could do that in my bathtub. That's amazing.

## NG

We have a series of questions having to do with the detailed mechanisms by which vaccines elicit immune responses. Do you have a complete accounting of what cells take up the RNA and then express the protein?

## DW

We've done extensive analyses, both in mice and in macaques. The issue is, from sensitive enough measurements, just about every cell takes up the RNA and makes protein. The issue was how much. If you do a cross-section of tissue, the dendritic cells are blaringly bright. They make a ton of protein. The surrounding cells make less. T cells make just about nothing. Fibroblasts, keratinocytes, you can see a little bit of stain, but they're not very bright. So, the dendritic cells are making blaring amounts of protein. And they're the central player in initiating new immune responses.

## NG

So, is there any spread from the local site of immunization, or is it pretty much limited to the area where the injection takes place?

## DW

What actually happens, and we're writing this up right now, is if you inject a decent dose into a mouse, so 10 micrograms, and then look at its lymph nodes 6 hours later, just about every lymph node in the animal will have loaded lipid nanoparticles (LNP) being taken up by dendritic cells. So, the LNPs are 80 nanometer particles, essentially viruses. They distribute through the entire body, and they home to dendritic cells and lymph nodes, to the liver, to the spleen, and a few other organs. So, to me, it's really the LNPs are traveling, not the dendritic cells, from the site of injection.

## MML

There's been some noise recently about advantages in humans to immunizing repeated doses of vaccine on the ipsilateral side of the first or on the contralateral side. Do you think that bigger humans would have a different distribution than your small mice?

## DW

So, the big difference is if you give mice a human equivalent dose, and I don't know what that is, but it's low, you don't see LNPs everywhere. But you see them through the entire lymph node chain on the draining side. Whereas if you give protein antigens, you see one lymph node. The draining lymph node. The first one is the only one. The LNPs spread to the entire chain. They don't spread to the whole body because there's likely not enough, and we can't detect it.

## NG

That's quite interesting. In terms of the spike protein vaccines, is the protein that's being produced secreted or expressed on the cell surface or both?

## DW

So, the ones that we worked with Moderna, BioNTech, Pfizer, Thailand, and a couple of others—those are all cell surface trimers. We've done comparisons of secreted trimers with trimerization motifs, and they work just as well. I don't know which is better. Functionally, they make the same immune response. And I think people, especially with all the craziness out there, don't want spike floating around the body.

## NG

My understanding is that CureVac has an mRNA vaccine, again focused on the spike protein of SARS-CoV-2, and they claim, as far as I know, that it was created without modifying the bases, which, as you know, has sort of been central to the conception of the vaccines for Moderna and Pfizer, BioNTech. What do you make of their findings?

## DW

If you read their phase 3 paper, it tells you why it failed. When Moderna and BioNTech did their phase 1 trials, the induced antibody levels were 3 to 5 times higher than patients that have recovered from disease. For CureVac's vaccine, the titers, I think, are like half or a quarter of what they were for patients. So, the phase 3 trial failed. They had less than 50% efficacy. Their claim was that new variants appeared. But I'm not sure I believe that. I think it was low antibody levels.

## NG

That's very valuable to know. Last in this series, what implications, if you've thought about this issue, do you see for self/non-self discrimination in the variable results that you described in your 2005 paper, and perhaps some subsequent work, in terms of the ability to silence Toll-like receptor (TLR) signaling, depending on what modification you were looking at.

## DW

We did it a as more of a mechanistic study, and we put Toll 7, Toll 8, Toll 3, then later RIG-I, MDA5 and NOD2 and others, into 293T cells or other cells that had no other sensors. And then we measured activation. And we found some modifications didn't activate at all; they induced no signaling. Other modified RNAs still did. So, A modifications induced normal signaling; U modifications didn't induce much of any signaling. So, our conclusion was that it's the Us in the RNA that are recognized as foreign. And when you modify them, that no longer occurs. [It is interesting to note] that there is one molecule that sees LPS. There are a couple that see DNA. There are 17 that see RNA. So, evolution has chosen how it wants to recognize foreign elements.

## NG

So, you can summarize it by saying that U defines you (i.e., covalently modified uracil, but not “naked” U, is a marker of immunological self).

## DW

Yeah.

## MML

The two major vaccines that utilize your technology to limit COVID were Pfizer and Moderna. Are you aware of major differences in terms of how they're made or what they live in.

## DW

So, there are minor differences. The untranslated regions are different. The coding sequence optimization is different. How they add their cap was done differently, but they both have natural Cap1. The lipid nanoparticles have a different formulation. The ionizable cationic lipids are different. We've done vaccines across many different sequences, many different LNPs; they all act the same way. They all have the same mechanism of action. Their utility is a function of how well the RNA is translated and how well it's delivered by the LNP. So, I see them as more improvements to the process, not different technologies.

## NG

Is it the LNPs that account for the difference in cold chain requirements?

## DW

Yeah. It's complicated because the pKa of the ionizable lipid is calculated, usually 6.4 to 6.7, as being optimal. But that's a pKa in an aqueous solution. If you measure the pKa in a lipid solution, it's about 2 to 3 points higher. RNA doesn't survive well in alkaline environments; it's degraded. So, depending on what the lipid pKa of the ionizable lipid is, that may determine breakdown of the RNA. There are also lots of other reasons—instability and other components—that determine the survivability at 4 degrees.

## NG

That instability that you just cited at higher pKas for RNA, is that purely chemical? Or is that enzymatic?

## DW

No, it's purely chemical. The basic environment degrades RNA.

## NG

Very interesting.

## MML

You've mentioned, and we sort of got to this a little bit earlier, about the paradoxical effects of immune sensing for your protein expression strategy. So, how'd you figure out how much was enough in the human system and how much was too much?

## DW

It was purely done in phase 1 clinical trials. We determined in mice, what was the smallest amount that we could use, and it was a function of the delivered protein. Some vaccines, we could give .01 micrograms, others needed 1 microgram. In humans, it was purely a phase 1—what people could tolerate. And they gave them the highest dose that they could tolerate.

## MML

And would you expect that this same relationship would take place irrespective of the sequences of the message?

## DW

The sequence of the message is important for how much protein is produced. And we find that protein production is a predictor of potency.

## NG

I don't know if you teach, but I do, and if you talk to students about what is responsible, what are the factors that influence immunogenicity? The standard immunology textbook focuses on self/non-self discrimination. Some sources will emphasize what is called “danger,” as opposed to non-self. How do you view that area? Or do you have any thoughts about that conceptualization issue?

## DW

So, when I grew up and trained in immunology, everything was self/non-self. And then Charlie Janeway and others came along with the inflammation hypothesis. My current feeling is that likely both are occurring. I think for immune recognition, inflammation is more important because you need to stimulate the immune system to respond. And non-self will do that, but it'll do it slower, it takes different types of cells, it takes [adaptive] immune cells to do it well. The innate immune system recognizes inflammation and responds immediately. So, I think for the initiation of immune responses to foreign pathogens and foreign elements, the inflammation is probably what I would lean towards.

## MML

How potent are the T cell responses, class I and class II restricted, after immunization with COVID vaccines?

## DW

They're variable. Part of it is dependent on the antigen. Part of it is dependent on the LNP. We've done head-to-head comparisons in macaques using adenovirus compared to mRNA. And with some antigens, the mRNA has a more potent T cell response. We know from the COVID vaccines, that it's the T cell responses that are protecting people from getting very sick and dying. The antibody responses prevent infection. But with all the new variants, the antibodies don't work as well anymore. And we keep re-boosting to try and re-focus them, but it's really the T cell responses that protect people from serious disease.

## MML

So, that's the classic paradigm that we taught our students. Antibodies protect against infections and T cells protect against morbidity of disease. So, would you see that there's a rationale or a goal to add some other viral elements into the COVID-19 vaccines to enhance the diversity of T cell responses?

## DW

Certainly. The issue there is that when you add multiple RNAs—we've added up to 20 so far in a vaccine—each antigen is produced at a fraction of the total. So, if you have 2 RNAs, you get half as much protein. So, if you start adding in other proteins, you'll get less spike protein and you'll get less antibodies. So, it becomes a balance.

## NG

Do you think it's possible to construct a COVID-19 vaccine based on mRNA that would be capable of yielding sterilizing immunity?

## DW

I'm not sure. I think when the Wuhan spike was around and hadn't turned into variants yet, we saw sterilizing immunity in those first people vaccinated. And then the variants appeared, and that was lost. So, I think it's certainly possible, but the problem, of course, is that the variants are appearing so quickly and all over the world. I don't know if we'll ever be able to do that. We're taking a different approach. We actually started this spring of 2020, even before we had a vaccine. We started working on pan-coronavirus vaccines. We submitted it to the National Institutes of Health (NIH), and the reviewer said it was not needed and didn't fund it. That was a separate argument. Back then, you had 3 coronavirus epidemics that occurred in 20 years, so there were going to be variants. We thought the pan-coronavirus was the way to go. We've got one now going into phase 1 clinical trials. So, it may not give sterilizing immunity, but it may prevent against pandemics or severe infection.

## MML

What do you think about a COVID-19 vaccine that targets mucosal sites?

## DW

We're working on that. Others are working on that as well. One of the big issues is that, in general, LNPs are toxic to the lungs. We can give a mouse 30 micrograms, we can give them 90 micrograms IV, and they tolerate it. But if we give them 5 micrograms inhaled, they don't do well. So, there's a lot of toxicity. Companies have developed variants that have less toxicity. There's one in a clinical trial for replacing CFTR in cystic fibrosis. We and others are using similar ones to try either intranasally, orally, or inhaled immunizations to induce mucosal immunity.

## MML

In the toxicity, is it the nasal mucosal, tracheal, or alveolar [cells]?

## DW

The lungs bleed.

## NG

Is that inflammatory, or is it just sort of a chemical mechanism?

## DW

You can't tell, because both are occurring, but we don't know what came first.

## MML

And it's related to the lipid?

## DW

Well, we assume that it's from the lipid nanoparticle.

## MML

We touched on this before when you mentioned that the nanoparticles are taken up by a variety of cells, although most effectively by dendritic cells, antigen-presenting cells. Do you think that modifying the surface of the lipid particle, targeting a particular cell, will get you selective expression? And is that something that you're working on?

## DW

We've already published a few papers on that. We've been able to target the lung with anti-PE-CAM antibodies. [1] We can target the brain with anti-VCAM antibodies. [2, 3, 4] We can target T cells with a variety of markers. [5, 6, 7] We published last year that we could make CAR-T cells *in vivo* and cure a disease in a mouse. [8] So, instead of 10 days of half-million-dollar processing and a fancy lab, we injected RNA LNPs, and made CAR-Ts, and cured the mice. We published a few months ago that we could target bone marrow stem cells with about 60% gene editing efficacy. [9] And we could do secondary transplants and retain that level. So, we were targeting the repopulating bone marrow stem cells. All of those are moving forward into new therapies, new clinical developments. We have a big sickle cell anemia program, with the idea that someday we'll be able to go to Sub-Saharan Africa and the entire world, give people an IV injection of offthe-shelf LNPs, and cure their disease.

## MML

That's fantastic. So, do you think that you could use these methods to selectively target CD4 T cells containing latent HIV provirus and release them from latency?

## DW

They're in macaques right now, we'll know the answer in a few weeks.

## MML

Fantastic.

## NG

I don't know if you've heard of this, but there's an investigator at MIT named Kevin Esvelt, PhD, who had worked with David Liu, PhD, Harvard University, and then with George Church, PhD, Harvard University. And I think Esvelt was involved with Church in developing gene drive technology. So, he's a very competent researcher, but he is not an immunologist, as far as I know, or a virologist, and he put forth the notion on a podcast I heard that potentially pathogenic viruses in the environment [identified by surveillance programs], should not be subjected to experimental work. And you should be able to just look at the virus genome, pick out an antigen, make an RNA, pop it in a vector, and you have a vaccine. I'm curious what your reaction to that approach would be?

## DW

I think it's a bit conservative and a bit overbearing. I don't think we want to go around getting sewer water, sequencing it, and making vaccines to anything we don't know that's in there. We've got a program we've been doing with DARPA (Defense Advanced Research Projects Agency) for a few years, it's in clinical trials right now, that they call a 60-day cure. And their idea was that they wanted to be able to go to anywhere in the world where a new infection just appeared, that they had no idea what it was, and it looked like it was spreading locally. They would give us a tube of blood, and with that, we would sequence to see what the pathogen was, we would isolate B cells to make monoclonal antibodies, and then optimize them and encode them as mRNA LNPs and give them to the local population to prevent the spread of the disease. To me, that that's a more realistic and useful approach.

## NG

I wanted to ask one other thing about the LNPs, which is do you understand or is it understood how LNPs—my understanding is that they have an adjuvant effect—is it known how that is working, ie, through what sensors or signaling pathways?

## DW

We don't know the sensors. We've excluded all of the Tolls, the helicases, the inflammasomes, the NODs, all of the known and expected innate sensors.

## NG

That's quite interesting. And on a quantitative level—if it's possible to assess the magnitude of the effect—is that anywhere near the [extent of the] effect of the non-modified RNA inflammation?

## DW

They're very different. So, the non-modified RNA is typically a TLR7 or 8 with maybe some RIG-I and other helicases. It gives a focused Th1 profile. The LNPs have a completely unusual adjuvant activity. They induce T follicular helper (Tfh) cells, [and] they're Th1 biased. We've done measurements. In a typical alum or MF59 vaccination, 5% of the CD4 helpers are Tfhs. With mRNA LNPs, it's over 50%. So, there's just a huge induction of Tfh cells. And I think that's what gives such great antibody levels.

## MML

Can you identify an effect of the LNPs that is independent of the presence of an mRNA? Is it all due to the lipid? Your readout is dependent on your message, obviously, but is the pathway independent of the presence of RNA?

## DW

Yeah, so we took empty LNPs with nothing in them, added them to protein antigen, and saw the same thing.

## MML

So, there are a lot of people out there who are reluctant to get immunized for one reason or another. How do you think scientists should address vaccine hesitancy in the public?

## DW

You know, it's a very complicated question. And it's really new. When we were young, we always knew there were anti-vaccine people, and they weren't a big deal. Anti-vax people have been around since Jenner was immunizing people for smallpox. What's different now is it's turned into a political crusade. And the far right, for whatever reason, thinks that vaccines are an attack on their liberty, never mind society's importance in the world. It becomes personal freedom, and there are a huge number of reasons why they don't want vaccines. The big problem is, they're supported by their leaders, by the clergy, by politicians, by local leaders. And that's a problem because they're doing it for power. They're not doing it because they actually believe. Everybody in Congress got vaccinated, but a large number of them sit there and say, don't take the vaccine, it's bad for you. And they hire Surgeon Generals who try to make RNA vaccines illegal.

## MML

I didn't know that everybody in Congress had been immunized. Is it a rule, or was it personal choice?

## DW

We don't know that. It was a rule to be able to return to Congress, in the beginning, everybody had to be vaccinated. Everybody at Fox News was vaccinated.

## MML

Here's another question about careers. Neil and I both trained as physicians. You trained as a physician. Should every physician who's serious about a research career go to PhD school?

## DW

I don't think so. I work with a lot of incredibly talented, basic science, researcher physicians. They get their training in years of fellowship. And I don't think that's all that different than a PhD. I think the training is important. I don't think how you do it is critical.

## NG

I would say, from my observations that, as you say, there are many MD-only individuals who have done very basic research and made huge contributions. I was at Washington University in St. Louis for my postgraduate work, and there were a number of examples there, at University of Pennsylvania as well when I was there earlier in my career. But I think it matters on who the fellowship mentor is and whether they have an appreciation for basic science and fundamental questions. I'm not against people who just have a clinical focus, but I think if the PI trainer is more clinically oriented, they may not transmit some of what you need to develop a strong foundation and basic understanding of biomedical mechanisms.

## DW

I completely agree.

## MML

Irrespective of going to PhD school or not, do you have any advice for young scientists who are embarking on a research career now?

## DW

Advice is worth a cup of coffee, maybe less. I'm asked a lot, and I try to give reasonable advice. One of the big things is that the personalities of researchers range the complete gamut. But the focus, the approach, and the thinking are much narrower. And there are some people that just shouldn't be in research. If you can't tolerate frustration, you don't want to be in research. So, there are some attributes that it's important [to possess]. What I tell young people, and I talked to a lot of high school students, is that, if you enjoy science, if you enjoy exploring, if you're curious, then give research a try. If your interest is in doing the same thing over and over and over, then science isn't going to satisfy you.

## NG

There is some of that in science: doing the same thing over and over and over. But I take your point. In fact, have you ever heard of Hershey heaven? That was Alfred Hershey, who won the Nobel Prize with Luria and Delbrück and was involved in the early experiments showing that phage replication required DNA—I think that was one of his contributions. And he said that Hershey heaven was when you could do the same basic experiment over and over and continue to get really important results.

So, [switching topics], Michael is the founding editor of our journal *Pathogens and Immunity*, and I'm one of several senior editors. And the motivation Michael had for establishing this new journal, which is a very modest-sized journal, in terms of the volume of papers and people supporting it, is to really modify the landscape of biomedical publishing so that we have more sensible policies and practices that make it easier for researchers to actually get their work out and make sure it's of high quality. So, for example, one of our chief features is that we allow people to submit papers in any format that's reasonable, that's standard, without worrying about our format, and we only require formatting for publication if it's accepted, which saves a lot of wasted time in reformatting submissions. So, do you have any advice for journal editors like us to improve how science publishing works?

## DW

You know, I'm asked the same question by people who oversee NIH referees. It's an incredibly difficult job. And I really don't know how it works and how to make it work better. Looking back when Kati and I tried to publish our work 20 years ago, and nobody would publish it, we wished there was a journal that was open-minded. But you know, I understand the problems. A journal gets 10,000 articles; it can only publish 100. They have to have maneuvers, they have to have procedures, they have to have guidelines in order to publish. So, I don't know how you overcome things like that. I like BioRxiv because it gets the research out quickly. It's not peer-reviewed, but at least you know it's out there, and you know what's going on.

## MML

Well, timeliness is important. And our journal tries to respect that by making the submission process exquisitely simple. It takes, on average, fewer than 5 minutes to put your manuscript on our website, and we're open access, no charge to authors, and we pay our reviewers [for timely reviews]—we have of a lot of things that make it a little different from your standard journal. But hopefully, you'll hear more about us. And, frankly, having you talk to us today is a wonderful thing for our journal, and I think for our trainees who come to our website. But now I've got to move a little bit off target and ask you, what sort of things do you do outside of the lab? What are you interested in outside of RNAs and vaccines?

## DW

Unfortunately, because of how things have gone over the past years, most of those things have been lost. I just don't have time. In my old days, my wife would joke, when I got frustrated at work, I would come home and build something like a porch on the house or renovate a bathroom. During the summer, I used to like to kayak every day and just get out on the water and relax. Unfortunately, things have gotten so busy between meetings and travel and everything else, there's much less time for that sort of thing.

## MML

Well, then, let me give you an easy question to answer at the end. What's your favorite baseball team?

## DW

Well, so I have the issue that I grew up in Boston, and all of my favorite teams are Boston teams. I was at the 76ers game Monday night and met the captains and had to smile and say, Philadelphia is great.

## MML

Well, Drew, listen, this was fantastic. It was wonderful to talk to you. And really, it was so good of you to spend some time with us. Thanks a lot.

## NG

I greatly appreciated not just that you participated, but the depth of your answers.

## DW

Very happy to do it.

## NG

Thank you again for a truly insightful interview.

## SUPPLEMENTARY DATA

Supplementary materials are available at the *Pathogens and Immunity* website. Supplementary data may be provided by the authors to benefit the reader. Supplementary data are not copyedited and are the sole responsibility of the authors. Questions or comments related to supplementary materials should be addressed to the corresponding author.
