# Supplementary figures and images for: Interview with Drew Weissman, 2023 Nobel Laureate in Physiology or Medicine
Source: Pathog Immun. 2024 Apr 16;9(1):22–37. doi: 10.20411/pai.v9i1.698 (PMC11107418; doi:10.20411/pai.v9i1.698)

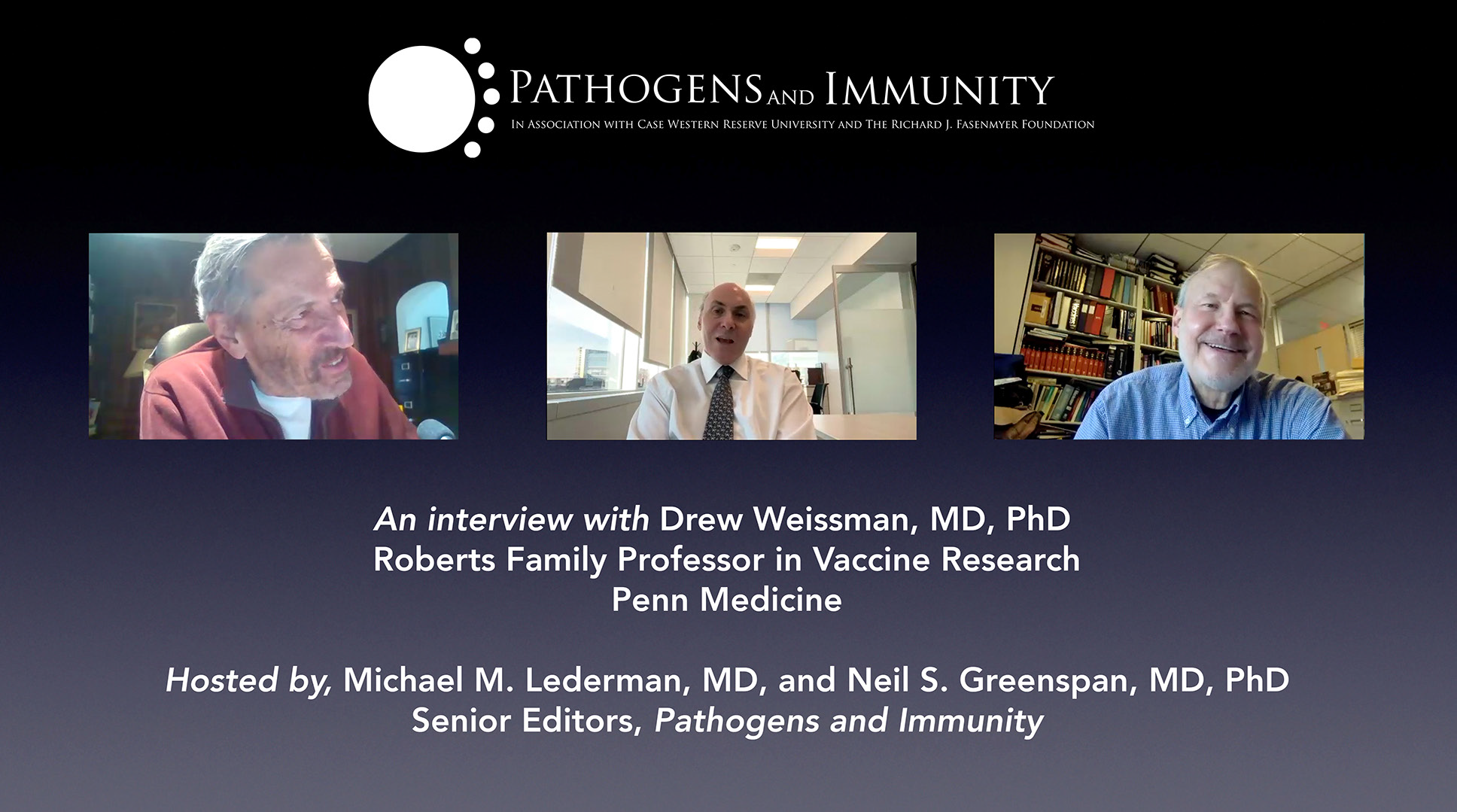

Supplement: Supplementary Video [file pai-9-022-s01.tif]
